# A Fast Electrical Resistivity-Based Algorithm to Measure and Visualize Two-Phase Swirling Flows

**DOI:** 10.3390/s22051834

**Published:** 2022-02-25

**Authors:** Muhammad Awais Sattar, Matheus Martinez Garcia, Luis M. Portela, Laurent Babout

**Affiliations:** 1 Institute of Applied Computer Science, Lodz University of Technology, Stefanowskiego 18/22, 90-924 Lodz, Poland; laurent.babout@p.lodz.pl; 2 Department of Chemical Engineering, Delft University of Technology, Van der Maasweg 9, 2629 HZ Delft, The Netherlands; m.martinezgarcia@tudelft.nl (M.M.G.); l.portela@tudelft.nl (L.M.P.)

**Keywords:** electrical resistance tomography (ERT), raw data processing, inline swirl separator, geometrical parameter extraction

## Abstract

Electrical resistance tomography (ERT) has been used in the literature to monitor the gas–liquid separation. However, the image reconstruction algorithms used in the studies take a considerable amount of time to generate the tomograms, which is far above the time scales of the flow inside the inline separator and, as a consequence, the technique is not fast enough to capture all the relevant dynamics of the process, vital for control applications. This article proposes a new strategy based on the physics behind the measurement and simple logics to monitor the separation with a high temporal resolution by minimizing both the amount of data and the calculations required to reconstruct one frame of the flow. To demonstrate its potential, the electronics of an ERT system are used together with a high-speed camera to measure the flow inside an inline swirl separator. For the 16-electrode system used in this study, only 12 measurements are required to reconstruct the whole flow distribution with the proposed algorithm, 10× less than the minimum number of measurements of ERT (120). In terms of computational effort, the technique was shown to be 1000× faster than solving the inverse problem non-iteratively via the Gauss–Newton approach, one of the computationally cheapest techniques available. Therefore, this novel algorithm has the potential to achieve measurement speeds in the order of 10^4^ times the ERT speed in the context of inline swirl separation, pointing to flow measurements at around 10kHz while keeping the average estimation error below 6 mm in the worst-case scenario.

## 1. Introduction

Common examples of two-phase flows are found in nuclear engineering [1], aerospace industry [2], and oil and gas exploration [3], which are the focus of this work. Oil is typically extracted from reservoirs with large quantities of water, with a global average of three barrels of water produced for each barrel of oil [4]. The oil–water mixture is conventionally separated by large tanks (e.g., API separators) that are expensive to install and maintain offshore. 

**Figure 1:**
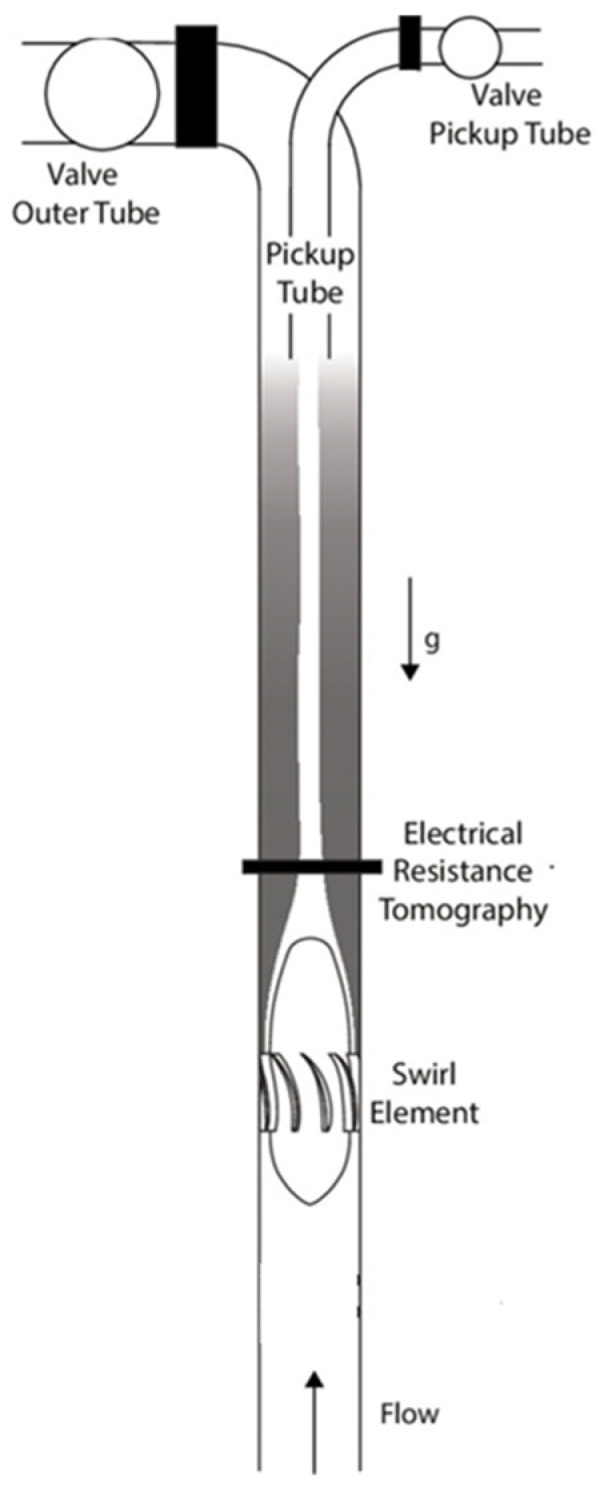
Schematics of the inline swirl separator with the sensor and pick-up tube considered in this research [7].

Inline swirl separators (ISS) have been developed in recent decades as a compact and cheap alternative to separate the oil–water mixture [5]. Inside the ISS, the mixture of fluids crosses a static swirl element and starts rotating. Due to centrifugal forces, the denser phase (water) is pushed against the separator’s wall and the less-dense phase (oil) is pushed towards the center of the system. The lighter phase accumulates in the center of the pipeline, creating a continuous core that is extracted by a pick-up tube placed by the end of the equipment [6], as shown in Figure 1.

Based on the flow properties upstream the separator, the light-phase core created by the static swirl element varies in size and eccentricity [8]. A potential approach to monitor the performance of inline swirl separators in real time is to measure the geometrical parameters (size and position) of the light-phase core created, which explains if most of the fluid is being captured or not when compared to the position and size of the pick-up tube [7].

Non-intrusive techniques to monitor the light-phase core are desirable to the oil industry since they do not introduce a pressure drop in the pipeline and do not require stopping the production for maintenance or replacement. In literature, numerous measurement techniques such as electromagnetic flow meters [9], optical cameras [10], ultrasound sensing [11], and electrical tomography [12] are widely used for the characterization of two-phase flows.

The advantage of tomographic techniques in relation to the other examples is that they give rich internal information of the process in a non-destructive/non-intrusive way, usually as images known as tomograms [13]. Based on the sensor design and electrode placements, the tomograms can give two (cross-sectional) or three-dimensional (reconstruction of a volume) information. Different operating principles are the basis for tomographic applications: electrical [14], microwave [15], ultrasound [16], gamma rays [17], and x-rays [18] are popular in a variety of research and industrial applications.

Electrical Resistance Tomography (ERT) is a promising non-intrusive measurement technique for real-time monitoring and parameter extractions of dynamic industrial processes [19]. ERT’s advantages include a non-hazardous application, low implementation costs, high measurement speed, portability, and straightforward implementation [20]. As a result, ERT has broad industrial applications in geophysical explorations [21], crystallization processes [22], materials investigations [23], and multiphase flows [24]. In the literature, one can come across studies that used current-voltage (CV-ERT) or voltage-current (VC-ERT) ERT instruments. While the traditional concept is based on the injected current–measured voltage scheme (CV), VC types can work with a broader range of conductivities and are conceptually simpler than CV-ERT systems [25]. 

The standard approach with tomography-based technology relies on a data-acquisition-image reconstruction scheme. Within the last two decades, numerous approaches have been proposed to cope with the two main challenges of electrical-based tomography: the non-linearity of the electromagnetic field and the ill-posedness of the inverse problem [26]. Many iterative and non-iterative methods are found in the literature to solve the inverse problem, such as Gauss–Newton [27], total variation [28], and linear back projection [29]. Iterative schemes have the advantage of more reliable image reconstruction while non-iterative schemes are faster to solve.

Both speed and spatial resolution are important to capture the relevant dynamics of the separation inside ISS in real time. Previous works based on the correction of the light-phase core size measured by the sensor via calibration against a camera were successful in increasing the accuracy of the ERT system for non-iterative schemes [30,31], and approaches relying on artificial intelligence and machine learning are also present in literature [32]. However, there is still a mismatch between the flow time scales and the temporal resolution of the ERT with the correction.

As an alternative to the image reconstruction, several studies [33,34] discuss the possibility of using raw data analysis (Tomometry) instead of tomograms to characterize two-phase flow measurements in a much faster way. Moreover, in many past studies the researchers have used electrical tomography raw data to characterize solid flows in hoppers [35], flow regime identification [36], and void fraction measurements [37]. However, there is a lack of literature regarding the possibility of extracting relevant geometrical parameters from the raw data. This study is part of this logic by proposing a method that considerably reduces the amount of data needed to estimate gas core characteristics for process control. For that purpose, this paper introduces the physical concepts behind the ERT measurements, successfully applying them to characterize the light phase core from the minimal number of measurements possible and directly from the measured data without the reconstruction step.

If one focuses on the process of interest of this work, namely Inline Fluid Separation, the topometric algorithms should have the following functional requirements:

- The algorithm should be able to give all the relevant information about the light phase core (e.g., size and position).
- If the visualization is needed, the algorithm should allow parametric reconstructions from the data. This also can open another dimension of innovative visualizations, for example, introducing Augmented Reality (AR) tools to inject process results in their real region of appearance.
- The algorithm should be able to calculate the required parameter online and should also be able to send the input to multiple streams, such as an input to the controller or the visualization tool mentioned above as fast as possible.

## 2. Algorithm Background

This paper suggests a general algorithm that reconstructs the electrically insulating core (oil/air) inside the inline swirl separator based on electrical current measurements at the pipe wall. A single plane of electrodes is used in the algorithm and the measurements required to reconstruct the flow distribution are made imposing an electric potential between a source electrode and all the remaining electrodes that are grounded, while measuring currents crossing the sink electrodes. To process the currents into a core size and position, the algorithm assumes a circular cross-section for the core at the measurement plane. In this paper, a VC ERT system with 16 electrodes is used as a proof of concept of the technique. 

**Figure 2:**
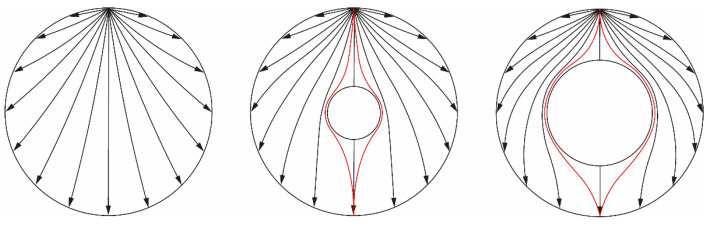
Estimated electric field lines inside the separator pipeline without an insulating core (**left**), with a small core (**middle**) and with a large core (**right**). The excited electrode (source) is always the top one. The black lines correspond to electric field lines reaching each sink electrode, since they are all at a lower potential than the source (grounded together). Additional electric field lines arriving at the electrode opposite to the source are presented in red.

The physics behind the algorithm can be easily understood by looking at the electric field lines created in the domain when one electrode (the top one) of the 16-electrode system is excited. Figure 2 presents three conditions where insulating cores of different sizes are placed in the center of the domain.

As seen in Figure 2 left, straight electric field lines arrive at the electrode opposite to the source in the absence of an insulating core. Since this is the smallest path possible that the field lines can take, the current measured at this sink electrode is maximum for a fixed potential. When an insulating region is present, as in Figure 2 center, the lines arriving at the opposite electrode (in red) get distorted since they must contour the non-conductive region. As a consequence, more energy is dissipated by the longer path, and the current observed in the sink electrode is smaller. It is clear in Figure 2 right that the lines get even more distorted when the size of the insulating region is increased, causing the current measured by the opposite electrode to be even smaller. 

It is natural after the explanation to assume that all the currents measured by the sink electrodes are reduced in the presence of an insulating core, and that this reduction observed in the current is connected to the size of the core: the bigger the core, the smaller the currents. The effect is best observable by the opposite measurement, which presents the most distorted electric field lines as indicated by the red curves of Figure 2. Therefore, it is expected that such measurement is strongly correlated to the core size.

**Figure 3:**
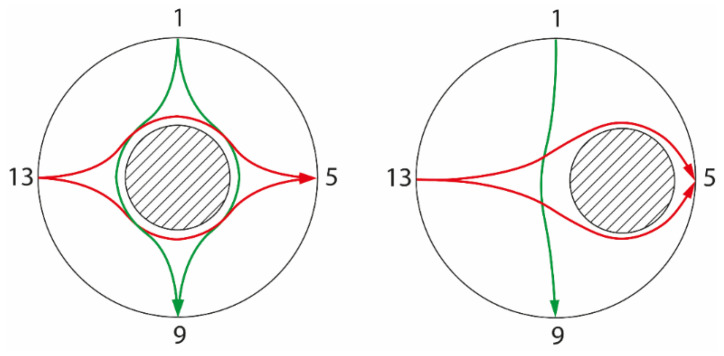
Electric field lines of opposite measurements. (**Left**): centered core, (**right**): core out of center. In green: electric field line when electrode 1 is excited. In red: electric field line when electrode 2 is excited.

As the core is centered in Figure 2, the system is symmetric in terms of the source electrode. For instance, if the electrode on the left side of the pipe is excited, the same images of Figure 2 are obtained but now rotated counterclockwise by 90^o^. However, in a real application the core is never perfectly centered in the domain and using different electrodes as source generates different images. Figure 3 illustrates what happens to different opposite measurements when the core is out of center. Note that the non-opposite electric field lines (in black in Figure 2) are omitted in the image.

Since the core is no longer centered, symmetry is lost and opposite measurements are distorted in different ways. The green line in Figure 3 indicates that when electrode 1 is excited, the electric field line reaching its opposite electrode is barely changed in relation to the non-disturbed configuration (Figure 2 left), such that the current measured in this case is close to its maximum value (obtained for the pipe without an insulating core). On the other hand, the red field lines contouring the core are formed when electrode 13 is excited; the field lines are more distorted than the respective centered case.

Looking at a single opposite measurement to predict the core size in this context would not work. If the measurement of Figure 3 considering electrode 1 is chosen, the core would be strongly underestimated by the small impact of the core in the electric field lines reaching electrode 9, and if the measurement considering electrode 13 is chosen, the core would be overestimated in size, as the lines are more distorted than the centered case. 

This difference when comparing opposite measurements can be explored both in the core size estimation and when tracking its position. In terms of the core size, taking the average between opposite measurements would cancel, at least in part, the under- and overestimations of the core size by the different, opposite measurements. In our setup, this is done by averaging the eight independent opposite measurements of the 16-electrodes system. In terms of radial position, the difference between the measurements is connected to an eccentricity of the core. If the core is perfectly centered, all the opposite measurements should be the same, independent of the electrode used as source. On the other hand, if the core is no longer centered, the opposite measurements will have different values. 

Taking Figure 3 as a reference, it is natural to think that the bigger the eccentricity of the core, the bigger the difference between the currents measured by the sink electrodes for opposite measurements. In this paper, the standard deviation between the eight opposite current measurements is used to compute the radial position (of the centroid) of the insulating core. In addition to the standard the deviation, the average current is also used in the computation of the radial position to account for the size of the core. This is a natural dependency: the electric field lines are distorted according to the core size and position. If a very thin core is present in the domain, the measurements will be almost always the same even if the region is strongly out of center, since a small core has a minor impact in the electric field inside the domain.

From the eight opposite measurements, both the core size (from the average) and the core radial position (from the standard deviation and average) can be determined. To be able to reconstruct the core inside the pipeline, the angular position of the core centroid is required. This is considerably more complicated than the remaining two estimations (core size and radial position) and it is done in two steps in the algorithm proposed in this paper.

First, the line of electrodes where the insulating core is present must be determined. This is relatively easy to do. Based on Figure 3, the opposite pair of electrodes aligned with the core presents the most affected electric field lines. Therefore, it is safe to assume that the core centroid is somewhere in the line between the source and sink electrodes where the smallest current is observed, in this case the line between electrodes 13 and 5. However, since the measurements are symmetric in relation to inverting the source and sink electrodes, the electrode closer to the core cannot be determined from the opposite measurements itself.

**Figure 4:**
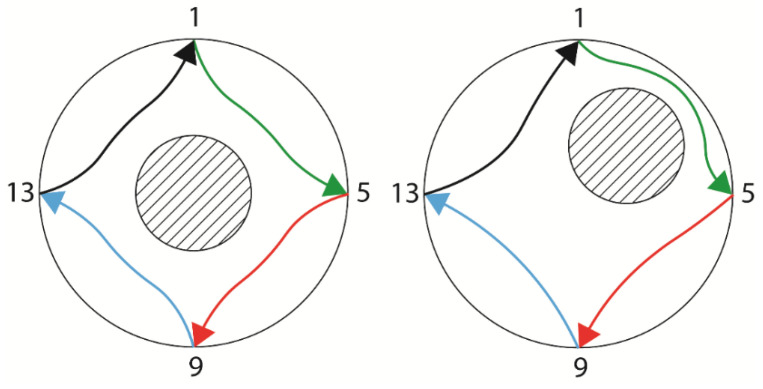
Electric field lines of the 90° measurements when the core is centered (**left**) and when the core is out of center (**right**). Each color represents one measurement.

To find the electrode closer to the insulating core, a new set of measurements must be performed. This is done with four additional 90° measurements, as illustrated in Figure 4.

As seen in Figure 4, the four 90° measurements are the same when the core is centered, but the lines are distorted in different ways when the core is out of center. While the current between electrodes 9 and 13 increases in Figure 4 right in relation to the Figure 4 left, as the electric field lines are less distorted by the out of center configuration, the current between electrodes 1 and 5 is the smallest of the four observed in the measurement, as those are the lines most distorted by the new core position. Therefore, the minimal current in the 90° measurements is used to set the quadrant where the core is present. 

The quadrant information allows one to filter the electrode closer to the core from the electrode pair with minimal current. The azimuthal position of the core is then given by the electrode number, with a precision of ± half the angle between two electrodes. Naturally, using more electrodes leads to a higher precision in determining the angular position of the core by the algorithm.

Although the angular position comes straight from minimal current values, the equations describing the size of the phantom and its radial position must still be obtained. This is explored in the next sections of this report.

## 3. Materials and Methods

This paper proposes the use of the raw data measured by the ERT system to reconstruct the main properties of the light phase core inside inline swirl separators. In this section, the ERT sensor electronics is introduced in Section 3.1, the calibration of the sensor based on phantoms is described in Section 3.2, and the flow loop used in the experiments is described in Section 3.3.

### 3.1. ERT Sensor and Measurement Electronics

The VC-ERT Flow Watch from Rocsole Ltd. (Houston, TX, USA), currently off the shelf, was used in the experiments. Sixteen stainless steel electrodes (M5 screws of 12 mm head) are used in the experiments, placed in a single plane embracing the 90 mm outer diameter-81.4 mm inner diameter PVC pipeline used in the inline swirl separator, as observed in Figure 1. The electrodes are installed in the pipelines via drilled holes and sealed using rubber sealing of 2 mm thickness installed in the inner side of the pipeline. A metal shield of 200 mm was installed around the sensor to reduce external electromagnetic disturbances in the measurements.

Inhouse-developed software TomoKis studio [38] was used for live image reconstruction and data acquisition. The acquisition unit saves all the 256 measurements of each frame at a frequency of 12 Hz. To match the impedance range of the signal with the target media, the electrodes were connected to the electronics using a signal conditioning unit. Coaxial cables type RG178 of length 2.5 m were used to connect measurement electronics and the sensor. For data acquisition, the device uses a particular sensing strategy where one electrode is excited and all the electrodes (including the excited one) are set for current measurement. 

### 3.2. Calibration Measurement 

Six cylindrical 3D printed ABS phantoms are used to obtain the link between the electrical currents measured by the ERT system and the core size and position. Cylindrical phantoms were chosen based on the application, where the light phase core is expected to be more or less circular with non-conductive property. To cover a wide range of core sizes, the non-conductive phantoms were printed with high precision with the diameters of 10 mm, 20 mm, 30 mm, 40 mm, 50 mm, and 60 mm. During this calibration, the phantoms are placed at different positions inside the pipeline to mimic eccentric light-phase cores in the separator.

**Figure 5:**
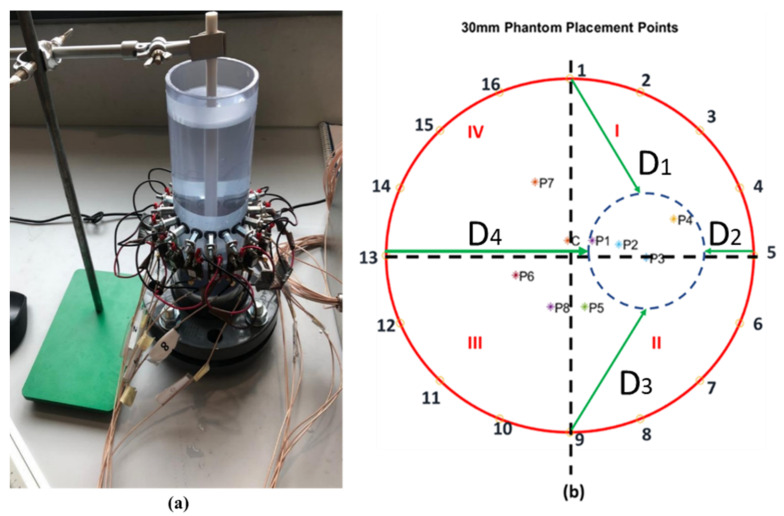
(**a**) Placement of 20 mm phantom inside the physical sensor (**b**) Procedure illustration for distance measurement from positions D_1_, D_2_, D_3_, and D_4_. The figure also shows the 9 positions used for the static measurements.

Each phantom is measured in the center of the domain and eight eccentric positions towards different electrodes. To keep track of the position of the phantom, the distance between its center and the electrodes 1, 5, 9, and 13 is measured in millimeters (mm) using a ruler. The approach is illustrated in Figure 5, where Figure 5a shows the phantom of 20 mm size placed in the center of the sensor and Figure 5b shows the four distances measured from the electrodes (D_1_, D_2_, D_3_, and D_4_).

The centroid position of the phantoms is recovered from the four ruler measurements considering a Cartesian coordinate system with origin in the center of the domain via a least-squares problem given by:

(1) $$F\left(x_{c},\;y_{c}\right)=\{\begin{array}{c} x_{c}^{2}+{\left(R-y_{c}\right)}^{2}-D_{1}^{2}\\ {\left(R-x_{c}\right)}^{2}+y_{c}^{2}-D_{2}^{2}\\ x_{c}^{2}+{\left(R+y_{c}\right)}^{2}-D_{3}^{2}\\ {\left(R+x_{c}\right)}^{2}+y_{c}^{2}-D_{4}^{2} \end{array}$$

where the *x_c_* and *y_c_* center coordinates of the phantom are obtained by minimizing *F*. Although two measurements would be enough to track the phantom, an overdetermined system with four measurements was considered to reduce human errors when measuring the phantom position.

Targeting a general approach that works independently of the conductivity of the domain (as long as the electric field can still propagate), a mixture of purified and tap water at different concentrations was used in the static tests. The sensor was filled with 1400 mL of water for each phantom, presenting five different conductivities ranging from 100 µS/cm to 474 µS/cm. Details of each water mixture are shown in Table 1.

### 3.3. Dynamic Measurements

The inline swirl separator of TU Delft was used in the dynamic tests to validate the proposed approach in a real-case scenario. The facility operates with air–water flows and is able to generate air flow rates up to 1000 L/min and water flow rates up to 350 L/min.

Air is injected in a vertical pipe with water flow 2.74 m (33.6 D) upstream the swirl element, allowing the gas–liquid flow patterns to develop in the section. The swirl element is 30 cm long and leads to an azimuthal velocity of around 2.5 times the bulk velocity at its outlet. A pick-up tube of 40 mm outer diameter (36 mm inner diameter) is extending down at the end of the pipe section to capture the gas for the separation process, as illustrated in Figure 1.

**Figure 6:**
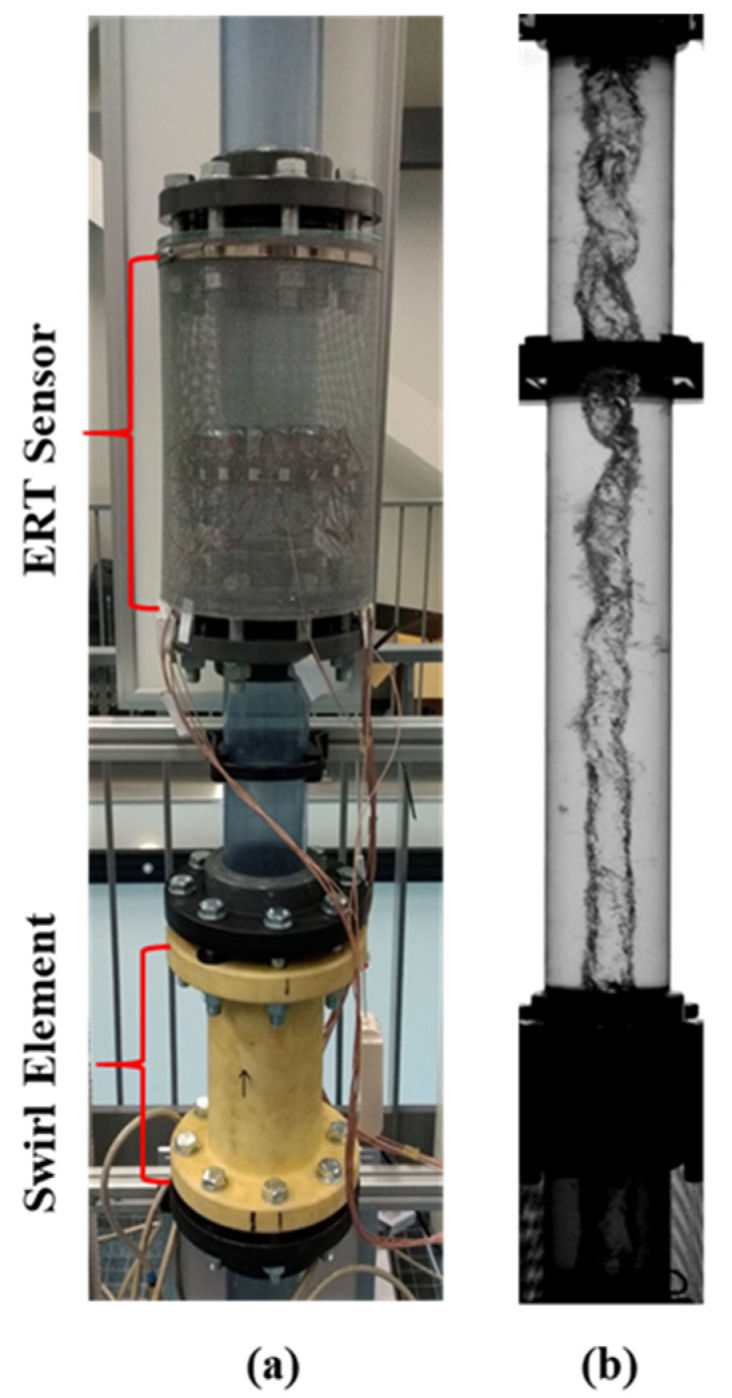
TU Delft Flow Test Facility (**a**) ERT position above the swirl element (**b**) High speed camera flow image, where the air core is present in the center of the device. The ERT shield can be seen in the bottom part of (**b**).

The ERT electrodes are installed 0.48 m above the swirl element, using the same 0.30 m long section used in the static tests. The entire region has an inner diameter of 81.4 mm. Flow conditions where a gas core above the ERT resolution (diameter greater than 10 mm) is formed were explored during the dynamic tests. In particular, the data obtained for a water flow rate of 160 L/min and an airflow rate of 100 L/min are explored in this text. The combination results in churn flow upstream of the swirl element, which causes the core size to continuously oscillate during the experiments. A picture of the region of the facility where the ERT electrodes are installed is presented in Figure 6a.

The flow taking place in the facility was recorded by a high-speed camera, Basler acA 1920 150 uc, at 60 fps. The same method described in [30] is used to recover the core size from the images and correct the observed values based on refraction theory due to the curved pipe walls. The images are processed in a window of 0.5 D starting 6 cm above the electrodes, inside the shield in the bottom of Figure 6b. Since a single camera view is considered and the gas core tends to stay around the center of the pipeline, only the core size is compared with the camera-based estimation during the dynamic tests.

## 4. Results and Discussions

### 4.1. Core Size and Tracking Algorithm

Although only 12 measurements are required to obtain the core size and position in this paper, the data acquisition of the ERT system used in this work cannot be easily changed and all the 256 measurements are still performed for each data frame, being later filtered into the 12 relevant values for the approach (the eight opposite electrode measurements and the four 90° turns). To make the quantities non-dimensional, the relations are performed for a normalized current:

(2) $$\Delta i_{m-n}=\frac{\;i_{m-n}\left(full\right)-i_{m-n}}{i_{m-n}\left(full\right)}$$

where i represents the current measured for the “*m* − *n*” electrode pair and full indicates the values measured by the ERT during the calibration with the domain filled with water. The normalized current varies between 0, when water is present in the pipeline, and 1, when pure air is in the domain.

**Figure 7:**
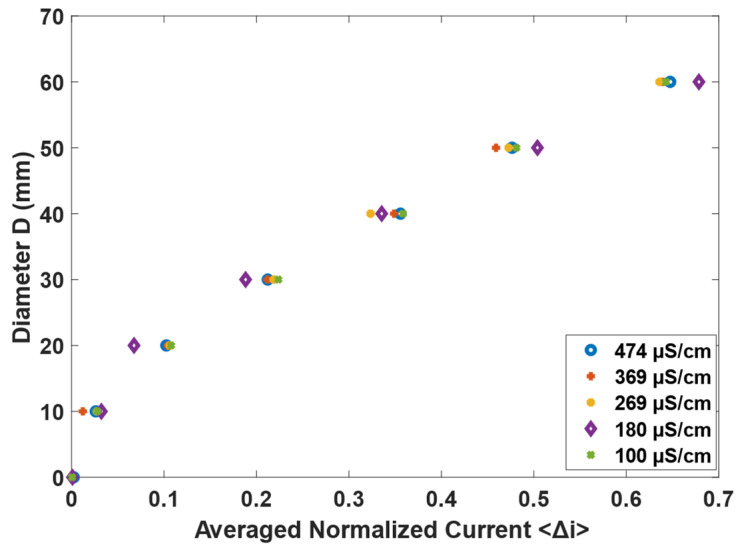
Relation between the normalized current and the phantom size for different water conductivities, represented by different symbols.

Since the full values of current already take into account the conductivity of the water, it is expected that $\Delta i_{m-n}$ is independent of the conductivity of the media. This is confirmed in Figure 7, where the normalized current is shown for different phantom sizes in the center of the domain, at different water conductivities. It is clear in the figure that there is a clear trend between the core size and the normalized current that is independent of the conductivity of the water used in the experiment, indicating that the equations developed in this paper are valid as long as the full measurements are used in the calculation of $\Delta i_{m-n}$. Therefore, the results obtained for the water mixture used in the flow installation for dynamic testing, i.e., 180 microS/cm are analyzed for the remainder of this paper.

#### 4.1.1. Core Size

As explained in Section 2, the current measured between opposite electrodes is connected to the size of the insulating region, which was visually confirmed in Figure 7. A few conditions must be fulfilled when defining a continuous expression for the core size based on the average normalized current. First, the core should be 0 when the normalized current is zero (since the full current is observed for the condition), and the core should be the pipe diameter when the normalized current is one. Among the different tests made, the condition is best fulfilled for:

(3) $$D=81.4{\;\langle \Delta i\rangle }^{0.6095}\;\left(R^{2}=0.99\right)\left(\mathrm{RMSE}=1.75\right)$$

where D is the diameter of the core, 〈Δi〉 is the mean normalized current difference ($\langle \Delta i\rangle =0.125\overset{8}{\underset{m=1}{\sum }}\Delta i_{m-n})$, and 81.4 is the pipe diameter in mm. 

**Figure 8:**
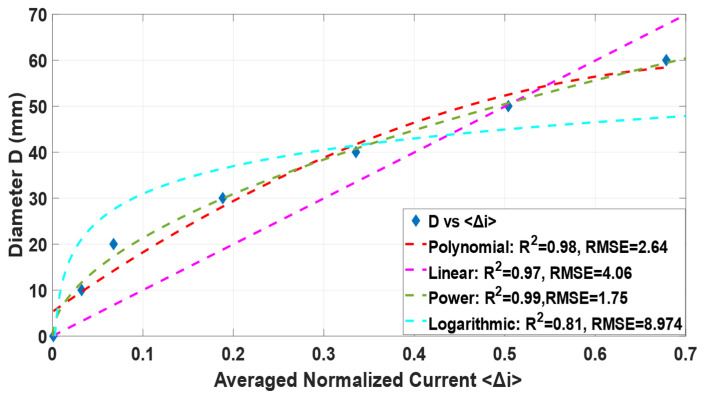
Comparison between (3) and the experimental dataset for the different phantoms at different locations in the pipe at 180 µS/cm.

Figure 8 presents (3) plotted for the phantoms at central positions. As predicted in Section 2, averaging the normalized currents reduces considerably the dependency of the position in the calculation of the core size from the current values.

The computation of the core size is summarized in the flow chart represented in Figure 10a.

#### 4.1.2. Radial Position

As stated in Section 2, the difference in the opposite measurements of current is connected to the phantom radial position and size. It was observed that the dependency on the size can be effectively eliminated by dividing the standard deviation of the current by the average current (connected to the core size by (3)). A possible explanation for this is that small cores out of center do not significantly distort the electric field lines, such that both the difference between the currents of opposite measurements due to eccentricity, represented by the standard deviation, and the decay in the current observed in relation to the full condition, represented by the mean normalized current, are small, and the effects cancel out in the ratio.

**Figure 9:**
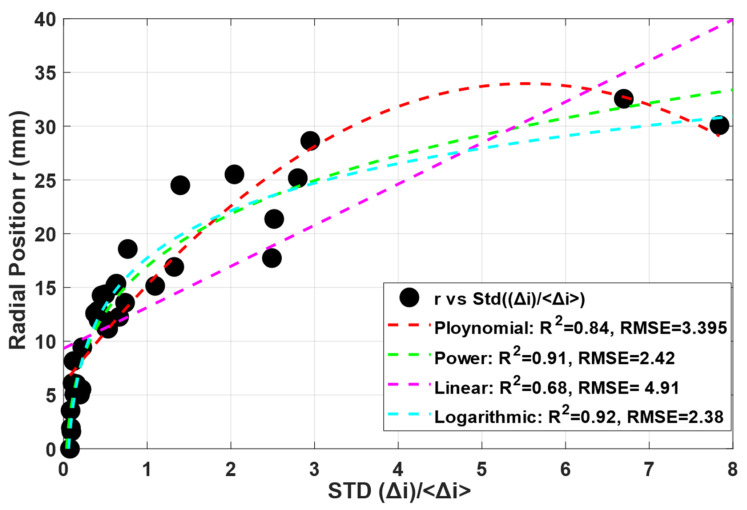
Fit obtained by plotting the standard deviation of the normalized current divided by its average against the radial position of the phantoms placed at different locations inside the domain. The blue dashed line represents the fit.

**Figure 10:**
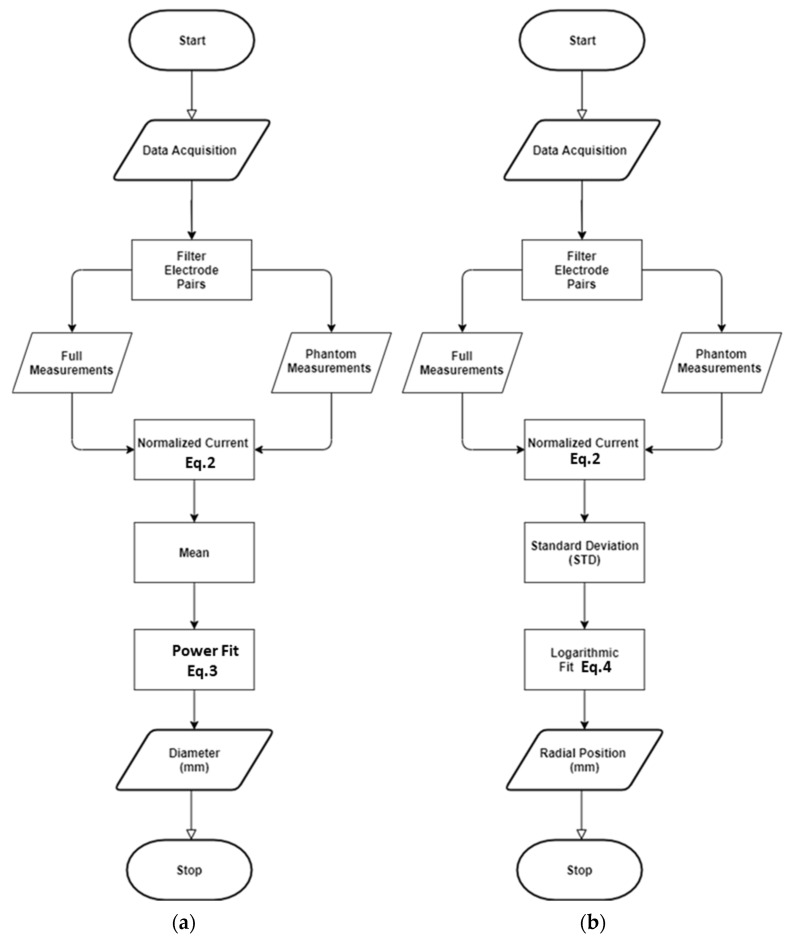

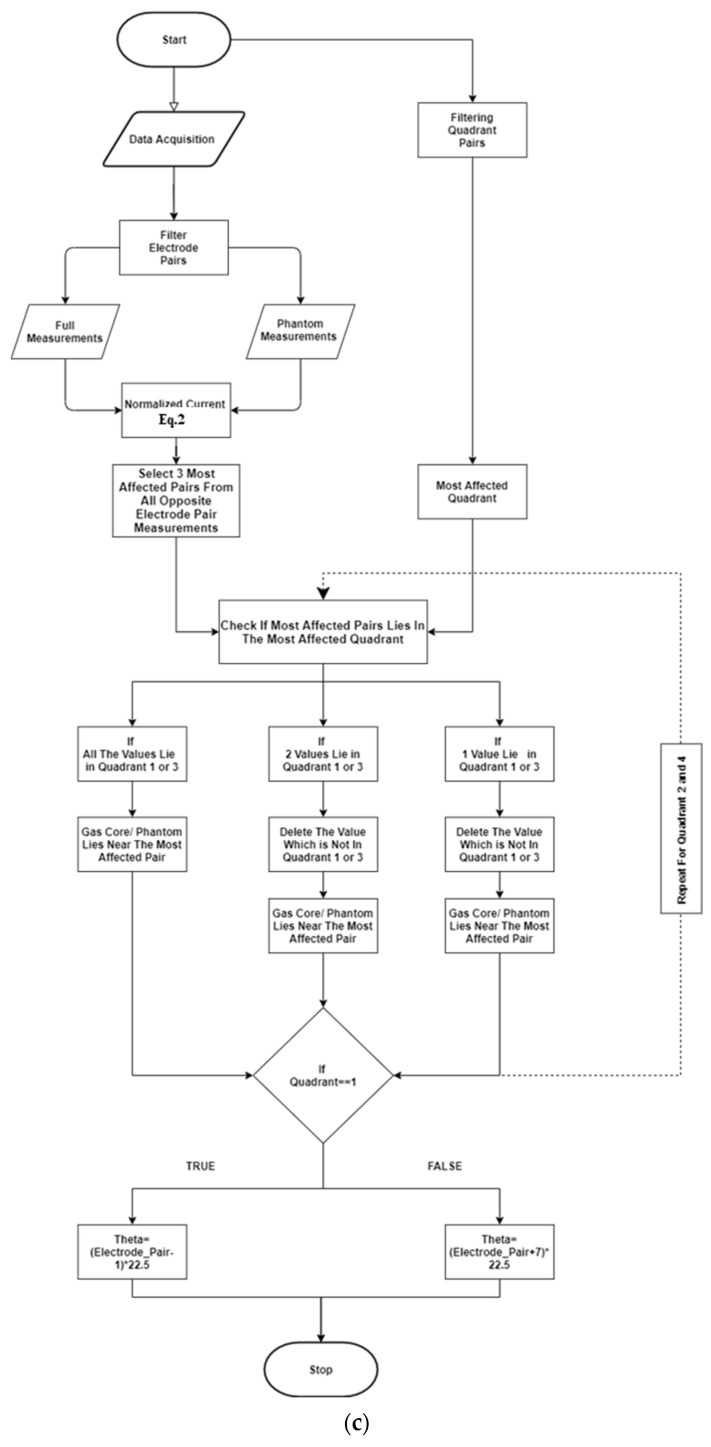
Algorithm flow chart: (**a**) diameter computing steps, (**b**) radial position calculation steps, (**c**) theta calculation routine.

During the experiments, it was confirmed that the standard deviation between the eight opposite measurements was close to zero when the phantom was in the center of the sensor, and it increased as the phantom was placed away from it. Different fits have been tested, and based on best R^2^ and RMSE values, the best fit has been adapted to estimate the radial position, which is illustrated in Figure 5. It was observed that when normalized by the average current, the standard deviation of the normalized current collapses around a logarithmic fitting expression:

(4) $$r=6.333\ln\left(\frac{std\left(\Delta i\right)}{\bar{\Delta i}}\right)+17.744\;\left(R^{2}=0.92\right)(\mathrm{RMSE}=2.38)\;\;\;\;$$

where r is the radial position of the phantom. The fitting curve and the original dataset for a water conductivity of 180 μS/cm (corresponding to a mixture with 60% of demineralized water as shown in Table 1) are presented in Figure 9. This radial position estimation is also summarized in the flow chart shown in Figure 10b.

#### 4.1.3. Angular Position

As explained in Section 2, the most distorted electric field line between opposite measurements is obtained for the electrode pair aligned with the phantom, and the electrode measurement is isolated from the pair using the additional four measurements of the current in 90° turns. The 90° measurements divide the sensing area into four quadrants, defined between electrodes 1 and 5 (I), 5 and 9 (II), 9 and 13 (III), and 13 and 1 (IV) as previously shown in Figure 5b.

From the physics of the problem, it should be possible to extract the information of the angular position of the core from the minimal currents of line and quadrant. However, due to noise or small differences between the currents, it was observed that sometimes unrealistic conditions are achieved, for instance, the line with smallest current was not crossing the quadrant with smallest current. As an alternative, some flexibility was required in the model when computing theta (*θ*), the angular position of the object in the polar coordinate system.

To handle the problem, the three most-affected Δi from opposite measurement pairs are considered instead of only the most affected one, and those are filtered based on the most affected quadrant. Since the turns of the quadrant are larger, it is expected that the measurement is more robust than the opposite measurements. To filter the electrode pair from the three possible options, the following logics are considered: if all the measurements match with the quadrant, the most affected electrode pair is used to reference the angle. If not, the one or two values which are not in the quadrant are discarded by assigning a null value and the decision is made based on the remaining most affected electrode pair. 

The measurements are made between electrodes *k* and *k* + 8, where *k* corresponds to the electrode numbers between 1 and 8. If quadrants I or II are the most affected, the electrode closer to the phantom is in the range 1 to 8, and if quadrants III or IV are obtained then the electrode is in the range 9 to 16. Once the electrode is selected from the combination between the electric field line information and the quadrant, the angle concerning electrode 1 in the clockwise direction is calculated by multiplying the electrode number by the angular distance between electrodes, leading to $\;\theta =\left(k-1\right)\;22.5^{{}^{\circ}}$ if the quadrant is I or II and $\theta =\left(k+7\right)\;22.5^{{}^{\circ}}$ if the quadrant is III or IV. The θ is calculated clockwise where $0^{o}$ is at electrode 1. 

The procedure is summarized in the flow chart of Figure 10c. 

### 4.2. Static Tests

The accuracy of the proposed algorithm during static measurements is compared with (i) the geometrical parameters (size and position of the gas-core) measured during the tests and (ii) image reconstruction results. For image reconstructions, the Gauss–Newton image reconstruction algorithm with Laplace regularization and the hyperparameter value of 0.5 was used in this research [32]. The circular 2D Finite Element Mesh (FEM) used in this research consists of 1024 triangular elements with medium vertex density and electrode refinement level of 2.

#### 4.2.1. Algorithm Validation

First, the diameter computed at each position using (3) was compared to the phantom’s actual diameter. Table 2 summarizes the estimated diameters obtained using water conductivity of 180 μS/cm. The positions of the samples referenced by C and P1–P8 are also shown in Figure 5b. 

The error in the estimation of the phantom size grows with its eccentricity, especially for phantoms below 30 mm in size. This is intrinsic from the method developed and connected to the approximation considered when estimating the phantom size from the average normalized current. As the distortion of the electric field is not exclusively dependent on the size of the phantom but also on its position, the location of the phantom has an impact on the current measured for the opposite electrode pairs. It seems, however, that the effect partially cancels out when taking the average between all the measurements, and the reported values of Table 2 show an error typically below 5 mm for the phantom size and a Root Mean Squared Deviation (RMSD) below 4 mm. The term “averaged maximum difference” is used for the average difference between the computed diameter and the actual diameter. The RMSD for 50 mm and 60 mm phantoms was not computed because of the availability of fewer data points when compared with the other phantoms; this can produce false results. 

The radial position (*r*) and the angle (θ) were compared with the values measured in the static test and summarized in Table 3 and Table 4, respectively. In the table, *r_r_* is the real radial position measured by the ruler, and *r_a_* is the radial position calculated using the algorithm. An average error below 3.5 mm in the radial location is obtained by the technique, with a maximum RMSD of 4.28 mm. It is notable that the RMSD values decay as the phantom size increases, which may be due to a high impact on the electric field when bigger phantoms are present in the sensing domain in comparison to the small phantoms or due to the small number of possible measurements for large phantoms, which quickly touch the electrodes for small eccentricities. This is also clear in Figure 9, which presents the fit and the experimental points adopted to calculate the radial position based on the current standard deviation, where it is notable that the points are much closer to the fitting curve for radial positions below 15 mm, while the points deviate from it for higher values. When observing Table 3, it is clear that $r_{r}>15\;\mathrm{mm}$ takes place only for 20 mm and 30 mm phantoms, in which the deviation is higher from the reference values. From the practical perspective, the insulating core is typically close to the center of the pipeline, and its small eccentricity can be effectively measured by the technique. 

The estimations of the angular position for both the real case (*θ_r_*) and using the proposed approach (*θ_a_*) are shown in Table 4. The table also shows the average absolute difference and the RMSD for all tested phantoms cases, like in the previous tables. One can first notice a high discrepancy between the *θ* values for all tested phantoms put in the C position, which is expected since the angle is ill defined at the location. For this reason, the values in the C position (shown in italics) are not taken into account in the statistical calculations. The table also shows an unexpectedly significant difference in the P1 position for the 30 mm phantom (shown in italics). The probable reason for this discrepancy is due to an electrode signal error during experimentation which results in considerable differences in both theta and radial position calculations. For this reason, this point is not taken into account when estimating the accuracy of the algorithm. In addition, the RMSD is not calculated for 50 mm and 60 mm phantoms as very few data points were available comparing to the other phantoms. Looking at the remaining values, one can see that the averaged absolute difference is ~24.50° and the RMSD ~20°. 

Concerning *θ*, it is crucial to keep in mind the precision used to calculate the value from the raw data. The angle between two consecutive electrodes is 22.5°, which limits the accuracy of the technique to ±11.25°. When compared to the results obtained, a small error when guessing the angular position of the phantom was obtained in the experiment. 

It is also worth combining the radial and angular positions from Table 3 and Table 4 to calculate the Euclidian distance between the real and estimated points. The Euclidian distance L can be calculated from the available quantities via:

(5) $$L=\sqrt{r_{a}^{2}+r_{r}^{2}-2r_{a}r_{r}\cos(\theta_{a}-\theta_{r}})$$

**Figure 11:**
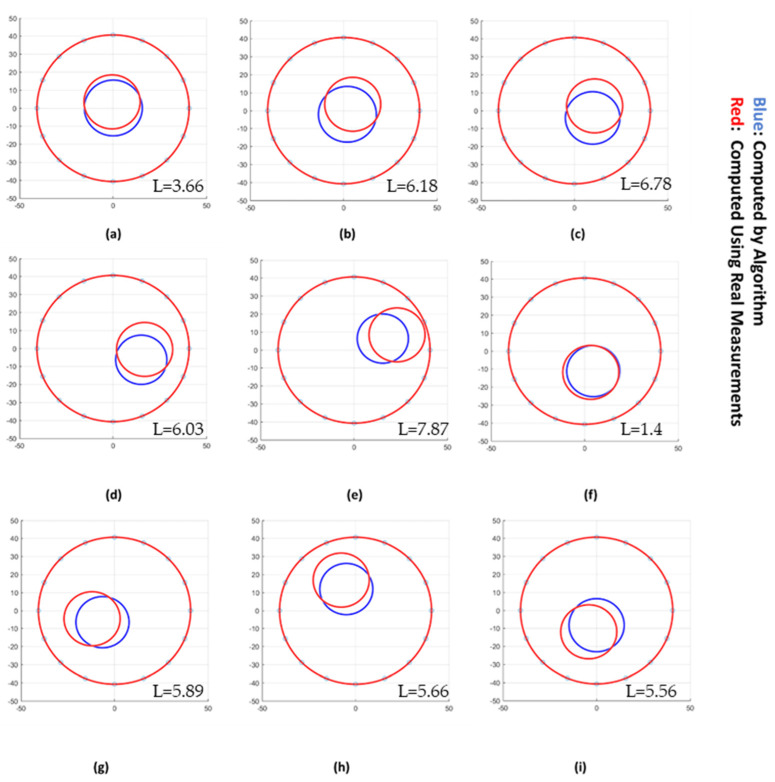
Comparison between the algorithm reconstruction (blue circles) and the real phantom characteristics measured using a ruler (red circle) for the point C to P8 (**a**–**i**) for the 30 mm diameter phantom. L values (mm) are also inserted in each figure.

In the case of the three smaller phantoms (20, 30, and 40 mm diameter), the average L values are 5.22 mm, 5.46 mm and 4.3 mm, respectively. The values are smaller for the two larger phantom specimens, i.e., 3.1 mm and 1.87 mm for the 50 mm and 60 mm phantoms, respectively. One still needs to keep in mind the small number of measurement points in these two cases. Figure 11 presents the reconstructed sizes and positions of the insulating region obtained by the authors’ algorithm for the points C and P1–P8, plotted together with the real position and size of the corresponding 30 mm phantom. The visual inspection of each case indicates a frequent error between the real and the predicted positions, which is considered acceptable when looking at the L values inserted as legends (average <6 mm). This error represented by L changes according to the size of the phantom, i.e., decreasing as the phantom size increases. This is an expected behavior, as large deformations in the electric field occur for larger elements, and the difference between the currents measured by distinct electrodes becomes more important, which generates better predictions.

#### 4.2.2. Comparison with Image Reconstruction

The algorithm results were also compared with tomograms obtained by electrical resistance tomography. Gauss–Newton (GN) image reconstruction algorithm and the same points shown in Figure 11 were chosen as references for the reconstruction. From the applied context, measurements with the core close to the center of the domain are the most important for the process. 

**Figure 12:**
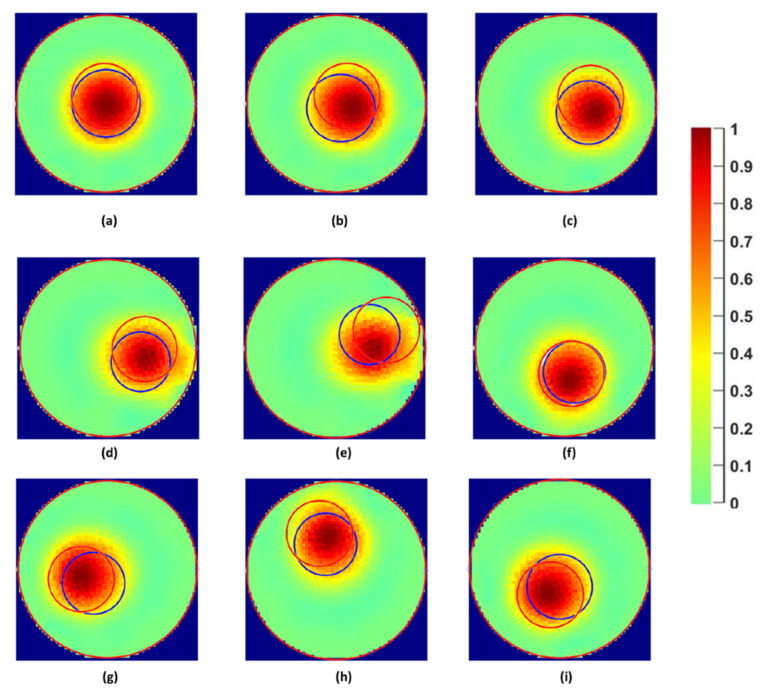
Experimental/real (red) and reconstructed (blue) diameter and position of the phantom superimposed on the traditional tomograms for the point C to P8 (**a**–**i**) for a 30 mm phantom. The colour bar is shown on the right side of the figure.

Figure 12 shows the comparison of the proposed technique (blue circles) and real phantom positions (red circles) with the tomograms obtained using the GN scheme based on the reconstruction parameters listed in the introductory part of Section 4.2. The figure shows that the visualizations obtained from the algorithm satisfyingly correlate with the tomograms in terms of size and position. Moreover, one can also notice a small discrepancy when the real positions are compared with the positions in the corresponding tomograms. Even though the reason needs to be further investigated, it shows that our proposed tonometry solution competes well with a standard image reconstruction approach in terms of accuracy. 

To numerically validate the comparison, the tomograms were processed using a graph-cut image segmentation method as described in [30]. The graph-cut method combines energy minimization and graph theory to detect sharp transition or object edges locally. From the segmented images, the radial position (*r*) and the angle (θ) were obtained and using (5), Euclidian distance L was calculated between the tomographic image and the *r* and θ recovered using the algorithm proposed in this paper. Table 5 summarizes this L result (L_3_) together with the ones corresponding to the two comparisons between the real cases and the reconstructions, i.e., L_1_ and L_2_. By looking at Table 5, one can notice a distance error of 3.68 mm, 2.83 mm, and 0.87 mm in the region of interest (center of the pipe) for the L_1_, L_2_, and L_3_, respectively. However, in the case of P4, a larger set of values, i.e., 7.87 in L_1_, 9.82 in L_2_, and 8.33 in L_3_, are obtained. This larger discrepancy, which is more pronounced for the circle reconstruction following GN image reconstruction + image processing (L_2_), is due to border effect. Indeed, the proximity of the phantom and the electrodes creates a signal distortion, which results in a shape approximation after image reconstruction. Nonetheless, for the remaining less-extreme positions (P1–P3, P5–P8), the distance between both reconstruction methods (L_3_) is about 3 mm on average, and the distance to the real position is around 6 mm. Considering the fact that the chosen classical image reconstruction method is also subject to localization error despite a longer processing time, the range of error obtained with the author’s approach is considered acceptable.

**Figure 13:**
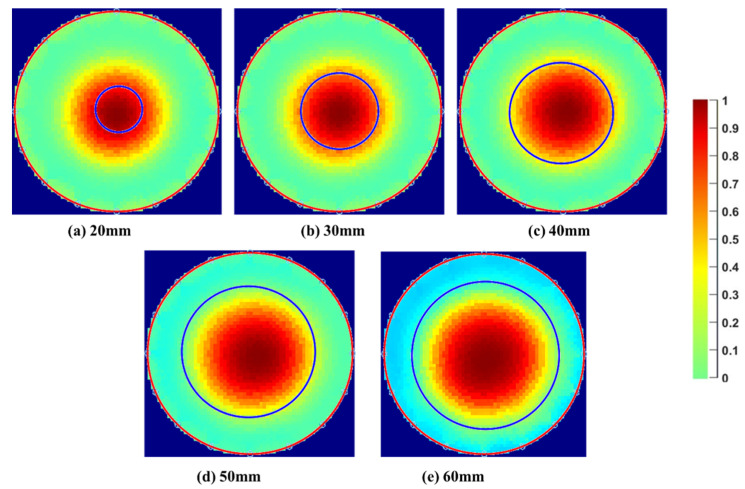
Diameter and position obtained using the proposed algorithm (blue) superimposed on the traditional tomograms in the low spatial resolution area. Case 20 mm to 60 mm phantom placed at the center of the sensing area. The colour bar is shown on the right side of the figure.

In the case of the diameter estimation, the discrepancy between the two techniques is accentuated in a low spatial resolution area (center of the pipe). Indeed, a considerable difference in the diameter estimation by the algorithm and the ERT image reconstruction is present, as shown in Figure 13. In past studies [30], the image reconstruction overestimated the diameter of the phantoms by 98% for small sizes and gradually improved with the increase in the phantom size. This discrepancy is mainly due to the ill-posed nature of image reconstruction algorithms. In this research, the tomograms were generated using optimal regularization parameters considering the conductivity of the water and the sensor shielding to avoid the effect of external noise. 

When comparing the time required to process the data, the tomograms required 98.6 s to reconstruct 2 min of data (1800 frames) using Intel Core i7 CPU and 16.0 GB RAM, whereas the algorithm can compute all the parameters in 0.12 s, 0.12% of the original time, corresponding to a core characterization done in ~67 µs/frame. The computational load for data processing is insignificant compared to the data acquisition rate of the ERT system used in the experiments, which is limited to measurements at 12 Hz. The same does not hold for the tomograms, which are considerably slow (55 ms per frame), which would limit the technique to around 18 Hz. This indicates that the algorithm could retrieve the flow quantities at higher frequencies if faster electronics are used. From the time required to reconstruct a frame, it is estimated that the algorithm proposed in this paper can measure the flow at a frequency of up to 15 kHz.

#### 4.2.3. Dynamic Tests

**Figure 14:**
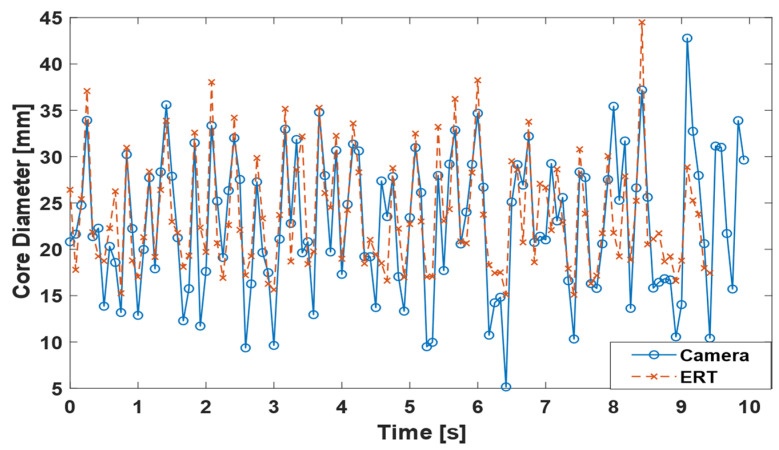
Time series comparison of the diameter between the ERT and the Camera at the point of maximum cross-correlation between the signals.

The comparison between the core size estimation from camera images and our proposed ERT-based approach is presented in Figure 14. The ERT operates at 12 Hz and the camera at 60 Hz. Therefore, during the comparison, the camera output is averaged every 5 frames, resulting in a 12 Hz signal that follows the ERT frequency. The two signals are then plotted at the same frequency and with the same number of points. The ERT has an internal delay in transferring the data to the loop, connected to transferring its data between computers via the UDP protocol, thus representing a delayed response concerning the camera when observing the core. To synchronize the two signals, Figure 14 is plotted considering a shift in the camera signal of +0.4167 s, as the beginning of the ERT signal corresponding to flow conditions measured before the camera was turned on due to the delay. Such a delay was obtained by cross correlating the two signals, where a clear peak appears for this lag. Figure 14 shows that the behavior captured by the camera is well represented by the ERT-based estimations, with a cross-correlation factor of 0.91. This indicates a good agreement between both modalities. In terms of core size average, both signals present similar values: 22.8 mm for the camera and 21.7 mm for the ERT. 

When looking more carefully into the signals, the ERT peaks of the core size have the same magnitude as the camera, indicating a proper capture of the core behavior. On the other hand, the valleys of the signals have different magnitudes, being shallower for the ERT than for the camera. The effect is a direct consequence of the low resolution of the ERT in the center of the pipe, especially when measuring small cores; the electric field lines present a small distortion for small cores, causing the normalized current observed in the region to be near the one for full calibration (i.e., only water). Consequently, cores below 10 mm cannot be detected, and cores between 10 and 20 mm are in the transition region, where their measurement is partially affected by the current region’s low sensitivity. 

The core monitoring in real time was validated by continuously sending the geometrical parameters computed by the proposed algorithm to a LabVIEW code via a dynamic link library (DLL). The DLL connects the TomoKis studio, filters the raw data, and computes the radial position, diameter, and azimuthal position based on the interpolation equations described above.

#### 4.2.4. Vision towards Smart Visualization

The central purpose of tomography technologies is to non-intrusively image the process into consideration. This is usually done on a separated device, e.g., a dedicated computer station where the reconstructed images are displayed. However, from the operating point of view, when direct visual inspection is necessary, it may not be the optimized situation to validate the accuracy of the reconstructed data by looking back and forth to the display and the installation where the process under investigation occurs. To overcome this drawback, a visionary approach could consider overlaying the display of the reconstructed data directly on the installation, preferably in real time. The proposed algorithm to parametrically reconstruct the gas core presented in this research gives hope that it is doable, provided that existing technologies can allow such a superposition.

The world is moving toward smart visualization using technologies such as virtual reality (VR), mixed reality (MR), and augmented reality (AR). In VR, an operator or an engineer can interact with and manipulate an object: a 3D computer-generated representation of a real-time object. Whereas in AR, the virtual objects are placed in the real world, users interact and visualize the object concerning the real world. Nowadays, various head-mounted devices such as Microsoft Hololens [39], Oculus Rift [40], and Samsung gear [41] are developed mainly to support AR applications. At the same time, competitive platforms such as handheld devices have proposed attractive alternatives to support AR in different contexts of use [42]. For instance, new generations of handheld Apple products support AR with cameras merged with LIDAR scanners. The AR is widely being used in gaming, furniture retail, medical, and the education sector. One of the main advantages of AR is the ability to support multiple users, allowing multiple experts to visualize and troubleshoot the process in real time. 

**Figure 15:**
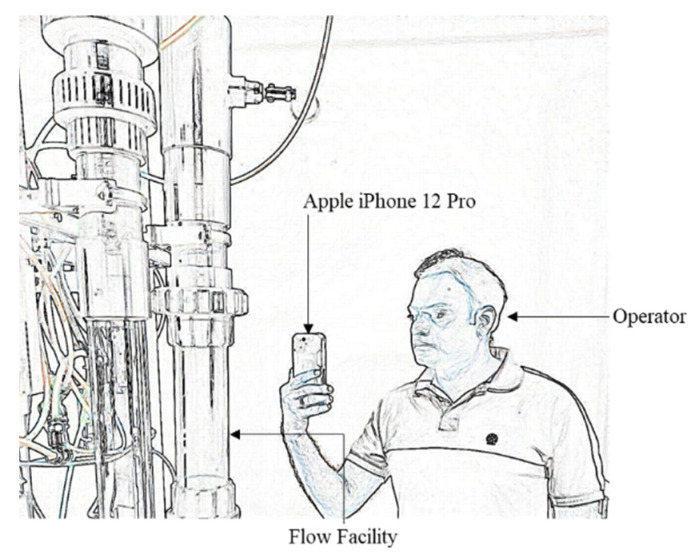
An example of operator visualizing the gas core using an AR handheld device.

Even with the vast adaptation of AR in other scientific sectors, process-based industries are not using AR for monitoring real-time processes. To fill this void in this research, a proof-of-concept AR visualization method based on the authors’ algorithm is proposed. The key purpose of proposing innovative visualizations is to allow users to visualize the sensor output in real time overlayed on the pipeline. The primary purpose is to simplify process monitoring by direct visualization, as close as possible to the area where the measurement of the process takes place. In addition, the solution could be of great help with flow installations made of opaque material where it is not possible to see what is happening inside. In such cases, these alternative ways of visualization can help the operators investigate the gas core’s geometrical parameters in real time. An example of such a case scenario is illustrated in Figure 15.

**Figure 16:**
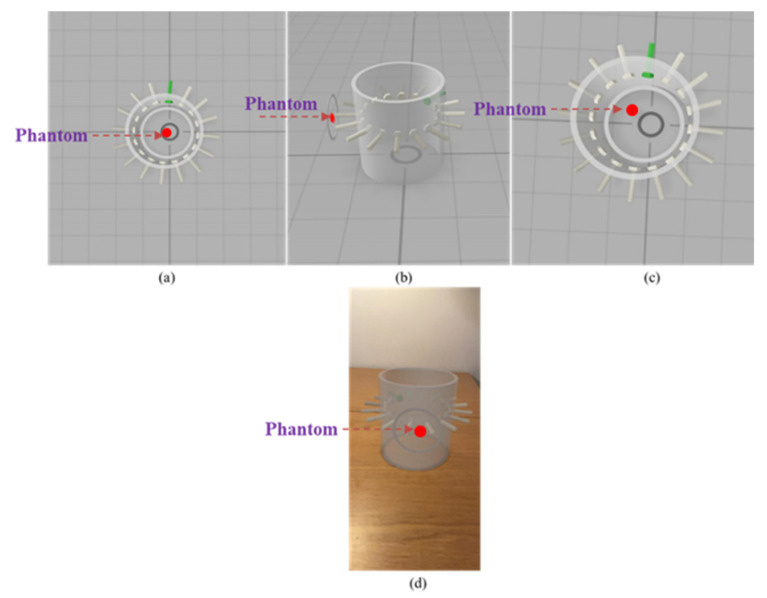
Smart visualization object displayed in Reality Composer application: (**a**) inside view, (**b**) superimposed view, (**c**) phantom position at P7, (**d**) AR testing on horizontal surface.

In this preliminary work, the usage of an Apple mobile phone (iPhone 12 Pro) with AR visualization method based on ARKit [43] is proposed, keeping the same style of visualization followed by the traditional tomographic image. The object was designed and manipulated using the following steps:

1. The object was created using the AutoCAD software. The main object consists of a virtual pipe and two circular objects where the outer circle represents the pipe, and the inner circle represents the phantom or gas core.
2. The object was transferred using a python script to Apple’s Reality Composer application. Reality Composer application contains various inbuilt libraries to manipulate and customize virtual objects sizes, shapes, and textures.
3. The main aim is to superimpose the object on the pipeline, so the anchor type ‘Object’ was chosen. This particular scanning type uses the LiDAR scanner to scan the pipeline where the user wants to see the object. The object is shown in Figure 16.
4. The transform parameters such as positions and size of the object were accessed in an Xcode11 IDE to manipulate the object parameters in real time.

In this research, only offline visualizations are proposed. The geometrical parameters computed from the static testing mentioned in Table 2, Table 3 and Table 4 were used to change the phantom position. The parameters were passed as an array with a delay of 1s between each consecutive point (P1–P8). 

Two views, namely inside and superimposed, were created as shown in Figure 16a,b, respectively. The top views show the movement of the gas-core inside the pipe structure, which is suitable for observing phantom movements in static testing. Whereas the superimposed view is created to show the gas-core on the pipeline structure. An optimal distance of 2.5 cm was chosen so that the image would not obstruct with the sensor screws outside the pipeline. Figure 16c shows the reconstructed position of the 20 mm phantom at point P7. The pipe object shows a green screw marking electrode number one, and in both views, the image object shows the actual position with respect to the position of the electrodes. Finally, the AR object was tested on a horizontal surface, as shown in Figure 16d. Object registration work is in progress to place the virtual sensor in its desired location, i.e., the pipeline where the process of interest occurs.

In these initial studies, both views were tested, and both show great potential towards bringing the AR visualization as a real time tool for process monitoring. However, there is a need for human computer interaction (HCI) studies to learn what kind of views a user or an operator prefers in different pipe materials. In addition, there is a dire need to study if the proposed AR visualizations cope with the fast data input streams and provide real-time and accurate visualizations of what is happening in the process under investigation, i.e., inline fluid separation.

## 5. Conclusions

In this paper, a tomometry strategy validated using an electrical resistance tomography system was proposed to monitor the size of the electrically insulating core created inside Inline Swirl Separators. The technique uses electrodes to measure a single plane of the flow and the results obtained in this paper indicate that:

1. The core size is well represented by the average current measured by electrodes installed opposite to each other in the pipeline. Moreover, using the equation proposed in this paper, a root means squared difference below 4.03 mm is observed when computing the phantom size.
2. The core eccentricity (radial position) is well represented by the standard deviation of the current measured between opposite electrodes for one frame divided by its mean value. The equation considered in this paper results in a root means squared difference of ~4.3 mm when estimating the radial position of the phantom.
3. The angular position of the insulating core can be successfully obtained from four additional 90° current measurements in addition to the opposite measurements, fully characterizing the core in 12 measurements using the 16 electrode system used in this paper.
4. The position of the phantom using the algorithm of this paper results in an average error of the distance between the real and the estimated positions of ~6 mm in the worst case occurring for the two smaller tested phantoms.
5. The proposed algorithm for core size and position estimation is suitable for dynamic monitoring of the inline separation, showing a potential measurement speed in the order of 10 kHz when measuring the core, in comparison to the speed in the order of Hz of the image reconstruction approaches.

## Figures and Tables

**Table 1:** Properties of the solutions tested during the calibration experiments.

Solution	Electrical Conductivity	Temperature
100% Tap Water	474 µS/cm	16.5 °C
20% Demin Water	369 µS/cm	16.5 °C
40% Demin Water	269 µS/cm	17.1 °C
60% Demin Water	180 µS/cm	18.1 °C
80% Demin Water	100 µS/cm	18.7 °C

**Table 2:** Diameter obtained for different concentric and eccentric cases.

**Phantom Diameter (mm)**	**Reference**	**10 (mm)**	**20 (mm)**	**30 (mm)**	**40 (mm)**	**50 (mm)**	**60 (mm)**
	Position						
	C	9.75	21.32	32.88	43.80	53.67	62.75
	P1	9.29	20.11	31.97	43.15	54.02	63.91
	P2	8.36	18.81	30.46	42.43	54.73	
	P3	6.27	16.43	28.97	42.24		
	P4	5.90	16.40	28.86	42.35		
	P5	7.95	18.14	29.78	42.84		
	P6	7.53	19.84	30.78	41.83		
	P7	6.33	15.75	28.97	41.81		
	P8	5.25	17.25	30.51	43.57		
	Average Absolute Difference	2.60	2.10	1.11	2.66	4.14	3.33
	RMSD	2.76	2.15	1.87	4.03		

**Table 3:** Radial position obtained for different concentric and eccentric cases. *r_r_* is the experimental radial position estimated using Equation (1). *r_a_* is the radial position calculated using the algorithm.

**Radial Position (mm)**	**Reference**	**20 (mm)**		**30 (mm)**		**40 (mm)**		**50 (mm)**		**60 (mm)**	
	Position	*r_r_*	*r_a_*	*r_r_*	*r_a_*	*r_r_*	*r_a_*	*r_r_*	*r_a_*	*r_r_*	*r_a_*
	C	1.61	1.27	3.56	0.20	1.93	0.93	1.06	0.90	1.39	1.24
	P1	5.03	4.56	6.03	2.58	5.52	4.61	3.99	1.13	2.68	0.77
	P2	11.24	10.51	11.17	10.59	5.53	4.61	2.68	6.10		
	P3	25.16	21.20	16.91	16.36	15.35	11.57				
	P4	28.63	21.52	24.50	16.67	9.44	4.82				
	P5	13.58	12.75	12.26	12.06	14.26	10.00				
	P6	8.15	1.71	12.83	9.10	14.26	9.50				
	P7	25.52	19.16	18.58	12.95	12.82	8.81				
	P8	15.15	15.08	12.59	8.21	6.11	0.50				
	Average Absolute Difference	2.93		3.30		3.31		2.14		0.68	
	RMSD	4.28		4.08		3.75					

**Table 4:** Azimuthal position obtained for different concentric and eccentric cases. The first row corresponds to the cases where the phantoms were placed very close to the center of the pipe. Maximum absolute difference and RMSD are calculated for the 8 other positions except P1 of 30 mm case. RMSD not calculated for 50 mm and 60 mm phantoms.

**Angular Position (°)**	**Reference**	**20 (mm)**		**30 (mm)**		**40 (mm)**		**50 (mm)**		**60 (mm)**	
	Position	*θ_r_*	*θ_a_*	*θ_r_*	*θ_a_*	*θ_r_*	*θ_a_*	*θ_r_*	*θ_a_*	*θ_r_*	*θ_a_*
	C	270	225	351.67	225	346.13	45	334.84	135	222.87	135
	P1	119.05	135	54	135	79.57	112.5	40.96	0	134.3	135
	P2	72.55	112.5	76.3	112.5	98.55	112.5	79.99	112.5		
	P3	79.5	112.5	91.7	112.5	97.96	112.5				
	P4	71.3	67.5	69.9	67.5	93.62	112.5				
	P5	147.12	157.5	164.39	157.5	170.31	157.5				
	P6	235.06	225	249.33	225	265.69	247.5				
	P7	331.05	337.5	335.5	337.5	336.24	315				
	P8	156.77	157.5	199.42	180	207.14	225				
	Average Absolute Difference	13.36		21.06		16.71		24.50		0.7	
	RMSD	20.02		19.89		19.72					

**Table 5:** Comparison of three Euclidean distances in the case of the phantom with 30 mm diameter size. L_1_: center distance between the real phantom position and the circle after parametric reconstruction (author’s approach); L_2_: center distance between the real phantom position and the circle after ERT GN image reconstruction (+image processing). L_3_: center distance between circles after parametric reconstruction and image reconstruction.

Points	L_1_ (mm)	L_2_ (mm)	L_3_ (mm)
C	3.68	2.83	0.87
P1	6.18	3.84	3.65
P2	6.78	5.28	0.95
P3	6.03	2.06	4.65
P4	7.87	9.82	8.33
P5	1.4	2.54	2.31
P6	5.89	3.73	4.47
P7	5.66	4.74	2.90
P8	5.56	4.95	4.71

## Data Availability

Not Applicable.
